# Revisiting the role of IL-1 signaling in the development of apical periodontitis

**DOI:** 10.3389/fdmed.2022.985558

**Published:** 2022-08-11

**Authors:** Kento Tazawa, Mariane Maffei Azuma Presse, Hisako Furusho, Philip Stashenko, Hajime Sasaki

**Affiliations:** 1 Department of Cariology, Restorative Sciences and Endodontics, University of Michigan School of Dentistry, Ann Arbor, MI, United States,; 2 Department of Pulp Biology and Endodontics, Division of Oral Health Sciences, Graduate School of Medical and Dental Sciences, Tokyo Medical and Dental University (TMDU), Tokyo, Japan,; 3 Department of Oral and Maxillofacial Pathobiology, Graduate School of Biomedical and Health Sciences, Hiroshima University, Hiroshima, Japan,; 4 Department of Translational Dental Medicine and Department of Endodontics, Boston University Goldman School of Dental Medicine, Boston, MA, United States

**Keywords:** obesity, diabetes, apical periodontitis, cytokines, interleukin-1 signaling, immune response

## Abstract

Apical periodontitis (AP) develops as a result of an immune response to pulpal bacterial infection, and various cytokines are involved in the pathogenesis of AP, with Interleukin (IL)-1 being considered a key cytokine. The role of IL-1 in the pathogenesis of AP has been well studied. It is known that IL-1 expression in periapical lesions correlates closely with the development of AP. IL-1 is a potent bone-resorptive cytokine that induces osteoclast formation and activation. Hence, inhibiting its signaling with IL-1 receptor antagonist (IL-1RA) results in a reduction in periapical lesion size. On the other hand, IL-1 is also a central cytokine that combats bacterial infection by activating innate immune responses. Therefore, a complete loss of IL-1 signaling leads to a failure to limit bacterial dissemination and consequently exacerbates AP. *In vivo*, IL-1 expression is tightly regulated and its signaling is modulated to optimize the immune response. Obesity causes systemic low-grade chronic inflammation and increases the risk of cardiovascular, renal, and other disorders. In experimentally induced AP, obesity significantly increases periapical bone loss, albeit the underlying mechanism remains unclear. Recent technological innovations have enabled more comprehensive and detailed analyses than previously, leading to new insights into the role of IL-1RA in regulating IL-1 signaling, and modulating apical lesion progression in obesity. In this review, we provide a brief overview of the function of IL-1 in AP development, with special emphasis on the latest findings in normal weight and obese states.

## Introduction

Apical periodontitis (AP) involves chronic inflammation and alveolar bone loss. Kakehashi et al. demonstrated for the first time that AP is caused by pulpal infection. Rats maintained in a conventional microbial environment developed pulp necrosis and periapical inflammation after pulp exposure; In a germ-free environment, the pulps remained vital without periapical bone destruction, and dentin bridges formed over the exposed pulp, demonstrating the capacity for tissue regeneration in the absence of infection (1).

In response to infection, complexly mixed immune cells migrate to the infected site. First neutrophils infiltrate, a followed by monocytes/macrophages, and subsequently by lymphocytes [T, B, and natural killer (NK) cells] (2, 3). These cells play critical roles in innate and adaptive immunity. Innate immunity comprises nonspecific responses that do not require prior sensitization to an antigen. Phagocytes are key to innate responses; neutrophils and macrophages engulf bacteria, and NK cells eliminate infected cells. Innate cells also produce inflammatory cytokines, which mediate immune and connective tissue cell activity (4–6). To eliminate pathogens and establish immune memory, the adaptive response activates antigen-specific CD4 + helper and CD8 + cytotoxic T cells, as well as B cells and plasma cells that produce antibodies (7, 8). The innate immune system lastly eliminate bacteria, apoptotic/dead cells, and debris. These responses are precisely regulated by the complex cytokine network.

Cytokines thus primarily protect the pulp and periodontal tissue from infection; however, cytokine-activated immune and inflammatory responses induce tissue destruction, particularly bone resorption (9, 10). Regarding bone resorption, Horton et al. firstly reported that immune cells can influence osteoclast activity in 1972. Osteoclast-activating factor (OAF), a powerful stimulator of osteoclastic bone resorption, was released from human peripheral blood leukocytes stimulated by the mitogen phytohemagglutinin, or by antigenic material present in human dental plaque (11). In 1985, OAF was subsequently purified to homogeneity and sequenced, and shown to be identical to interleukin-1-beta (IL-1β). It was later shown that *macrophage-derived IL-1 is a prominent mediator in developing bone destructive periapical lesions* (12–15). These and other basic studies on the interactions between the immune system and bone following pulpal infections have been important in establishing the field of osteoimmunology. These basic studies have provided a rationale of clinical research on IL-1/IL-1 signaling in AP and foundation for interpreting their outcomes (16–22).

Obesity is one of the most prevalent non-communicable diseases and predisposes to various disorders, including hypertension, type 2 diabetes mellitus (DM), dyslipidemia, and coronary heart disease (23, 24). The increased morbidity associated with obesity is a worldwide public health issue (25). Besides, obese people are more susceptible to infections than their non-obese counterparts as well to developing serious complications from common infections (26). AP is one of the most prevalent oral infectious diseases. In DM subjects, where obesity is the greatest risk factor, the success of root canal treatment is decreased, in teeth with AP (27, 28). Moreover, studies in the rodent diet-induced obesity (DIO) model have revealed that obesity promotes the progression and severity of experimental AP (29–31). However, the underlying mechanism(s) by which obesity alters the immune response in AP remain unclear.

As the background for future basic and clinical research, this mini review aims first to reaffirm the role of IL-1 signaling in the development of AP in the lean state, and then to provide new insights into the possible mechanisms underlying the expansion of periapical bone destruction associated with obesity, based on the latest experimental findings.

## IL-1 signaling is the central pathway in periapical lesion development

The IL-1 family comprises 11 cytokines: 7 pro-inflammatory mediators (IL-1α, IL-1β, IL-18, IL-33, IL-36α, IL-36β, and IL-36γ), and 4 anti-inflammatory cytokines [IL-1 receptor antagonist (RA), IL-36RA IL-37, and IL-38] (32). Each family member binds to a specific primary receptor which combines with co-receptors to transduce pro-inflammatory or anti-inflammatory activity. The primary receptors include IL-1 receptor type 1 (IL-1R1), IL-1R2, IL-1R4, IL-1R5, and IL-1R6. The co-receptors include IL-1R3, IL-1R7, IL-1R8, IL-1R9, and IL-1R10 (32, 33). IL-1α, IL-1β, and IL-1RA are the primary members that regulate the progression of periapical lesions, and their roles have been well studied. In contrast, the role of the other family members in the development of AP has not been systematically evaluated.

IL-1α and IL-1β are encoded by *IL1A* and *IL1B* respectively in humans (34). Both isoforms bind to IL-1R1 and show similar biologic activities, including immune cell activation (33, 35). IL-1 is also closely involved in both bone formation (36) and resorption (12, 15). IL-1 inhibits nodule formation by osteoblasts in a dose-dependent manner (36). IL-1 strongly promotes osteoclast differentiation indirectly by inducing the expression of receptor activator of NF-*κ*B ligand (RANKL; Tumor necrosis factor ligand superfamily member 11) in osteoblasts (37). IL-1 directly induces the fusion of mononuclear pre-fusion osteoclasts and enhances osteoclast function (resorption pit-forming activity) (38–40). Moreover, activation of NF-*κ*B promoted by IL-1 prolongs osteoclast survival (41, 42). However, IL-1α and IL-1β differ in several ways. First, species differences are found in their expression in periapical lesions. In rodent lesions, the predominant isoform is IL-1α rather than IL-1β (43, 44). In contrast, the protein level of IL-1β in human periapical exudate is double that of IL-1α (45). Furthermore, the bone resorption potency of IL-1β is 13-fold that of IL-1α in a rat assay system (10). Second, the expression level after root canal treatment is different. Following treatment, the level of IL-1β in the periapical exudates decreased, while the level of IL-1α increased. This suggests that IL-1α and IL-1β may play different biological roles in the healing process (45, 46). In this regard, a finding that bacteria-induced IL-1β and IL-1RI-myeloid differentiation factor 88 (MyD88) signaling are necessary and sufficient for efficient wound healing and tissue regeneration (47) is interesting. Third, the IL-1β cannot bind to IL-1R1 unless it is cleaved into its biologically-active mature form. Conversely, IL-1α precursor can bind to and activate the IL-1 receptor without proteolysis (48).

The expression level of IL-1 positively correlates to the extension of bone destruction and severity of AP. IL-1α mRNA and protein expression was identified in murine periapical lesions from the early stage of development, with increased levels found on day 7 after pulp infection (43, 44, 49). Higher levels of IL-1α and IL-1β were detected in human periapical lesions with severe inflammation than mild inflammation (50, 51). In periapical lesions, IL-1 is produced by various cells, including macrophages, fibroblasts, polymorphonuclear leukocytes, endothelial cells, osteoblasts, and osteoclasts in response to infection (44, 49). Among these cells, macrophages are the major source of IL-1. Macrophage-derived IL-1 plays a critical role in the periapical immunity. IL-1β and IL-1α are respectively 1000- and 75-fold more potent in stimulating bone resorption than TNFα or TNFβ (lymphotoxin) respectively *in vitro* (10). Besides, IL-1 neutralization significantly reduced bone resorptive activity in extracts from periapical tissue explants, whereas TNF-α neutralization had no effect (13, 15).

These studies focused on the bone-destructive effects of IL-1, but IL-1 also protects the host early after bacterial challenge. Antibody-mediated neutralization of both IL-1α and IL-1β leads to a failure to contain pulpal infection in male but not female mice, resulting in orofacial abscesses and sepsis (52). Ovariectomized mice also developed sepsis, but were protected by an estrogen implant. Accordingly, IL-1 signaling synergizes with estrogen signaling to prime phagocytic cells for enhanced anti-microbial activity resulting in infection localization. IL-1R1 deficient mice identically showed severe bone destruction and sepsis after pulpal infection (53, 54). Taken together, a severe deficiency of IL-1 signaling leads to poor infection control, dissemination of infection, and elevated bone destruction.

Subsequent studies using IL-1RA have confirmed the correlation between IL-1 and bone resorption. IL-1RA, produced by macrophages and monocytes (55), competitively blocks the action of IL-1. IL-1RA binds to IL-1R1 with equal or greater affinity than IL-1α and IL-1β but does not activate downstream signaling (34, 55, 56). IL-1RA has a significant impact by suppressing periapical lesion development. Stashenko et al. demonstrated a 14-day IL-1RA treatment inhibited lesion development by approximately 60% (57). Maintaining IL-1 and IL-1RA in balance prevents excess inflammation and bone destruction. Once this balance is upset, inflammation and tissue damage may deteriorate (58). To block IL-1-mediated bone resorption *ex vivo*, rat fetal long bones and mouse newborn calvariae require approximately 10-fold and 100–1000-fold IL-1RA to IL-1, respectively (59). In periapical lesions, the level of IL-1RA is more abundant than IL-1 (mean IL-1RA: IL-1β ratio = 128: 7). Interestingly, exudates from symptomatic human lesions contained a significantly lower ratio of IL-1RA to IL-1β than exudates from asymptomatic human lesions (22). Taken together, the local balance of IL-1 and IL-1RA is crucially important in the periapical lesion development.

## The cytokine network in periapical lesions centered on IL-1 signaling

Macrophages are major players involved in the cytokine network, and secrete various immunoregulatory mediators, including IL-1 (35, 60). TNF-α is another pro-inflammatory cytokine expressed by macrophages (61) and increased in periapical lesions (44, 49). TNF-α promotes IL-1 secretion from murine resident peritoneal macrophages *in vitro* (62) and increases osteoclastogenesis by upregulating RANKL (63, 64). However as noted above, TNF-α itself is not much bone resorptive as IL-1 isoforms, and TNF-α deficient mice exhibited similar periapical lesion size to wild-type controls (65).

The role of type-1 T-helper (Th1) cytokines [Gamma interferon (IFN-γ), IL-12, IL-18] and Th2 cytokines (IL-4, IL-6, IL-10) on periapical bone destruction has also been evaluated. IFN-γ, IL-12, and IL-18 potentiate pro-inflammatory signaling (66–68) and their expression is increased in periapical lesions (43, 69, 70). IFN-γ modulates macrophage-derived IL-1 expression, but its effect is not consistent. IFN-γ promotes secretion of IL-1 from LPS-stimulated human macrophages *in vitro* (71), whereas suppresses IL-1 in mouse RAW 264.7 macrophages (72). IL-12 induces Th1 cell development, and IL-18, with IL-12, activates established-Th1 cells to produce IFN-γ. Thus, IL-12 and IL-18 are considered pro-inflammatory cytokines that facilitate type-1 responses (67, 73). However, previous studies demonstrated that gene knockouts of IL-12, IL-18, and IFN-γ all exhibited similar lesion sizes as wild-type controls (65, 74). Recombinant IL-12-infused wild-type mice also showed similar bone resorption to controls. The findings with IFN-γ were not confirmed in another study which reported that IFN-γ-deficient^(−/−)^ mice presented with periapical lesions larger than those in wild-type animals (75). The expression level of IL-1 in periapical lesions was unchanged in these mice (74). Taken together, these results indicate that none of these cytokines has a non-redundant function in mediating periapical bone resorption.

IL-6, another macrophage-derived cytokine, was also detected in inflamed periapical tissue (76, 77). Its expression was found to be transiently increased on day 14 after infection and decreased in the chronic phase (43). IL-6 is a well-known pro-inflammatory cytokine, promoting bone resorption *via* osteoclastogenesis (78–80). Recent research has demonstrated that IL-6 also has anti-inflammatory effects by promoting macrophage IL-1RA secretion (81) and bone-forming effects by enhancing osteoblast differentiation (82–84). Previously, the protective role of IL-6 in periapical lesions was showed *in vivo*. Bone destruction was significantly increased in IL-6^−/−^ mice versus in wild-type mice (69, 85). IL-6 antibody-mediated neutralization also increased bone resorption compared to untreated controls. In IL-6^−/−^ mice, increased bone resorption importantly correlated with osteoclast count and IL-1 expression in periapical lesions, and inversely with anti-inflammatory IL-10 expression (69).

Both IL-4 and IL-10 are increased in periapical lesions (69). IL-4 is an anti-inflammatory cytokine playing pleiotropic roles in inflammation (86, 87). IL-10, a potent anti-inflammatory cytokine produced by regulatory T cells (Treg), macrophages, dendritic cells, Th 2 cells, and Th1 cells, among other immune cells (88–90). However, IL-4 and IL-10 have different anti-inflammatory effects on macrophages. In macrophages stimulated by oral pathogens, recombinant IL-10 inhibited IL-1α production, whereas recombinant IL-4 had no significant suppressive effect (91). Consistent with these *in vitro* findings, IL-10^−/−^ mice exhibited significantly greater infection-stimulated bone resorption than wild-type mice, as well as markedly elevated IL-1 production in periapical inflammatory tissues (91). In contrast, there was no difference in periapical lesion size between IL-4^−/−^ and wild-type mice (75, 91).

IL-17 is a pleiotropic cytokine produced by Th17 cells that induces a myriad of pro-inflammatory mediators (92). The expression of IL-17 was increased in infection-induced periapical lesions (65) and was significantly higher in symptomatic versus asymptomatic lesions (93). IL-17 induces human macrophages to produce and secrete pro-inflammatory cytokines IL-1β and TNF-α *in vitro* (94). IL-17A^−/−^ mice were resistant to periapical lesions versus wild-type controls (65). However, IL-17 receptor type A-deficient (IL-17RA^−/−^) mice conversely exhibited significantly increased bone destruction and inflammation. The expression of IL-1 was significantly upregulated in IL-17RA^−/−^ lesions *in vivo* and IL-17RA^−/−^ macrophages *in vitro*. The lesion size of IL-17RA^−/−^ mice was decreased by IL-1β neutralization (95). IL-17A utilizes two IL-17 receptors, and IL-17RA has four ligands (96), therefore, this system must be meticulously dissected to comprehend these data. Nevertheless, IL-17RA signaling likely plays a protective role in periapical lesions *via* IL-1 signaling and neutrophil priming.

Table 1 Summarizes the effect of cytokine or receptor deficiency/neutralization on periapical lesions. Although it is difficult to evaluate the effect of each cytokine because of their complex interactions (97), above reviewed experimental models suggest that anti-inflammatory cytokines such as IL-10 and, to a lesser extent, IL-6, are dominant and have non-redundant functions, compared to inflammatory cytokines in the immunomodulation of AP. In addition, the positive correlation between the IL-1 level and lesion size implies IL-1 is a principal cytokine in periapical lesion expansion and a useful biomarker for assessing inflammation.

## The impact of obesity and diabetes mellitus on periapical lesions

It is now widely accepted that obesity causes systemic low-grade chronic inflammation (98). As noted above, obesity increases the risk of severe inflammation (26), and predisposes to the development of postoperative and nosocomial infections, as well as serious complications of common infections (98, 99). Obesity also increases the risk for severe symptoms and poor prognosis in viral infections, including coronavirus disease 2019 (100). In the oral cavity, obesity correlates with the prevalence and severity of periodontitis (101). Deshpande et al. reported that obesity worsens all gingival index, probing depth, gingival recession, and clinical attachment levels than non-obese patients (102).

Diabetes, as an obesity complication, also has negative effects on AP. Diabetes decreases the success rate of endodontic treatment in teeth with AP preoperatively, and increases the risk of post-treatment tooth loss (27, 28, 103–105). According to previous *in vivo* rodent studies, obesity significantly increases bone destruction in experimentally-induced AP (29–31). As discussed in the following section, several potential mechanisms underlying obesity-induced inflammation have been proposed, but the actual mechanism is not yet fully understood.

## Potential mechanism of obesity-exacerbating periapical bone destruction

Many studies provide evidence that obesity alters immune responses. In obesity, macrophages significantly accumulate in the white adipose tissue (106, 107); and the phenotype of accumulated macrophages possess a pro-inflammatory M1-polarized state, whereas resident macrophages in lean mice have a pro-resolving M2 phenotype (108–111). The M1-dominant adipose macrophages likely develop an inflammatory milieu (112). The circulating levels of pro-inflammatory cytokines, including TNF-α, IL-6, and IL-1β was elevated in obese subjects (113, 114). Chronic exposure to these cytokines potentially causes insulin resistance resulting in hyperglycemia (115, 116). In addition, the serum levels of adipose tissue-derived cytokines, adipokines and adiponectin are also altered in the obese state. Obese adipose tissue increases inflammatory adipokines, including leptin, resistin, visfatin, IL-6, TNF-α, and monocyte chemoattractant protein-1, while decreasing anti-inflammatory adipokines, including adiponectin, omentin, IL-10, and IL-4. The dysregulation of adipokine production may alter cellular immune function and contribute to chronic low-grade inflammation and disease pathology (117–119). Obesity also increases the populations of activated CD4+ and CD8+ T cells in adipose tissue (120) and significantly reduces circulating T_reg_ cells (121–123) which may sustain low-grade chronic inflammation. Furthermore, obesity induces thymic involution and convergent T cell repertoire, impairing impaired immune responses and increasing the risk and severity of infections (124).

As noted above, the effects of obesity on immune function are manifold. However, it remains unclear how obesity is associated with the expansion of periapical bone destruction. Therefore, our group examined possible pathways involved in bone loss in obesity using bulk-mRNA next-generation sequencing analysis. Comprehensive gene expression analysis revealed that, among a total 15,029 expressed genes, only 51 were differentially expressed in periapical lesions in DIO-B6 mice versus lean controls. Among them, *Il1rn* encoding IL-1RA was remarkably down-regulated (Log2 fold change = −1.18, False Discovery Rate (*q*-value) = 0.0002). At the same time, *Il1a*, but not *Il1b*, was also decreased (−0.994-fold, *q* = 0.046) (31). These results suggest DIO impairs IL-1RA-dependent homeostatic suppression of IL-1 signaling, at least in the local environment.

**FIGURE 1:**
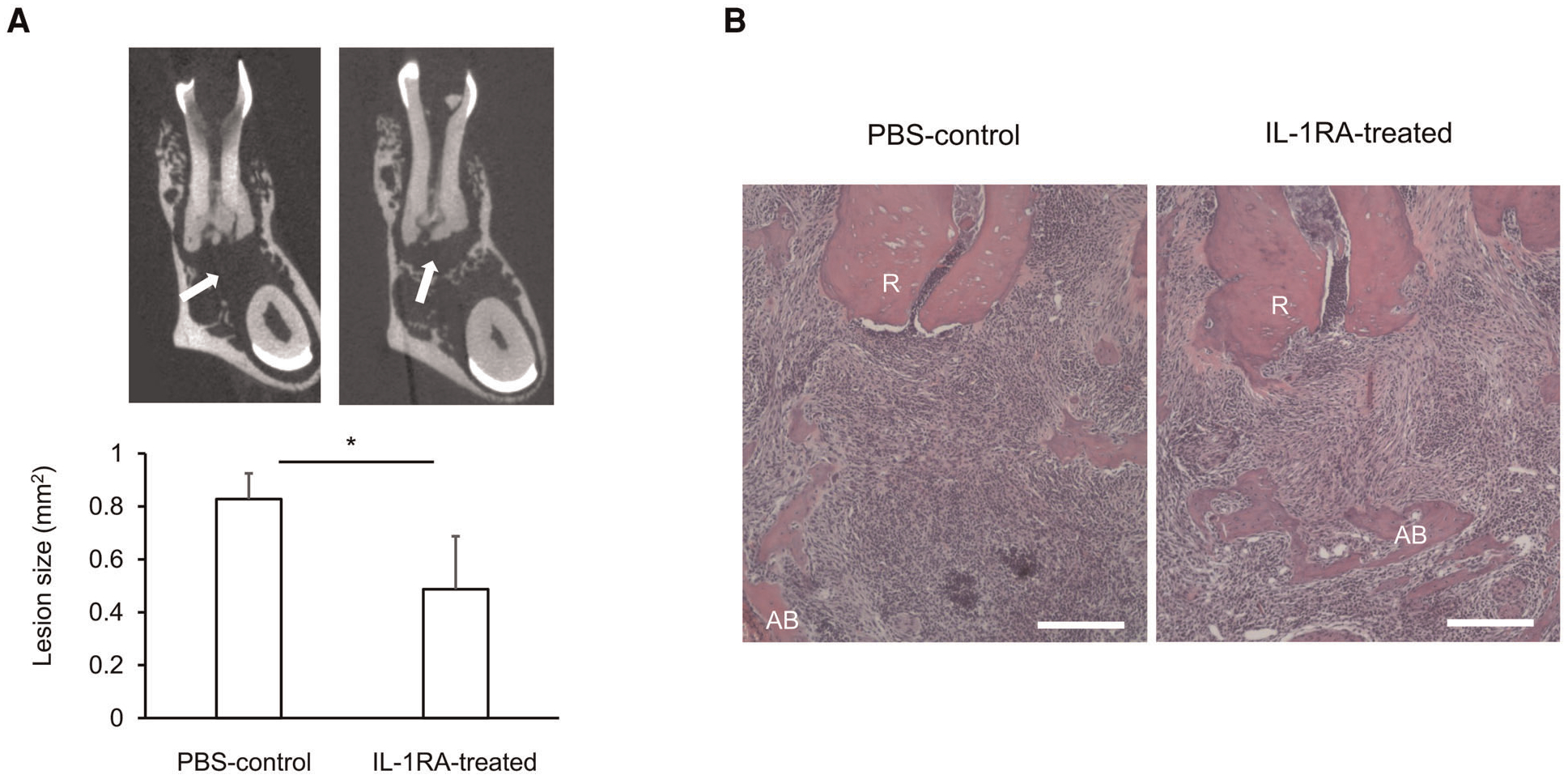
(A) Representative microCT images and periapical lesion size in phosphate buffered saline (PBS)-control and IL-1RA administration on day 42 after endodontic infection. Values are mean and SD; *p < 0.05. Arrow: periapical lesion. (B) Histology of periapical lesions on day 42 after endodontic infection. Representative images of hematoxylin and eosin (HE) staining for each group are shown. PBS, phosphate buffered saline; AB, alveolar bone; R, dental root; Scale bars = 200 μm.

Systemically, significantly increased IL-1 serum levels (114, 125) likely contribute to worsening of insulin resistance under obese conditions (116). However, given the lack of significant changes in the expression of IL-1 signaling genes, including NF-*κ*B, in AP (31), systemically increased IL-1 may have little effect on AP. Interestingly, IL-1RA serum levels are also elevated in obesity (126). However, the concentration of IL-1RA is likely insufficient to block the effects of elevated IL-1. Indeed, administration of IL-1RA improves insulin sensitivity in animal models of obesity (116), suggesting IL-1RA-dependent homeostatic regulation of IL-1 signaling is not fully functional in obesity. We therefore examined if a decrease or loss of IL-1RA contributes to obesity-associated periapical inflammation by IL-1RA administration in infected DIO-B6 mice. Remarkably, periapical bone destruction was inhibited by 41.2% by IL-1RA (Figure 1A, *p* < 0.05). Histological analysis revealed that IL-1RA-treated mice showed less inflammatory cell infiltration and well-developed fibrosis (Figure 1B). These results indicate that inflammation was down-regulated by IL-1RA, and that the lesion was composed mainly of mature granulation tissue compared to the immune granulomas in controls. Therefore, immunomodulation by IL-1RA is likely important for the control of AP, even in obesity.

Appropriate regulation of IL-1 signaling according to the host and infection status may lead to an optimal immune/inflammatory response in terms of timely onset/resolution and adequate host defense. In the first section, we explained that excessive IL-1 and its signaling cause exacerbation of AP in the non-obese state. At the same time, IL-1RA homeostatically regulates IL-1 signaling, suppressing excessive IL-1-mediated responses. In the second section, we described that obesity dysregulates IL-1RA-dependent homeostatic IL-1 signaling regulation and causes chronic elevation of inflammation, tissue destruction, and prolonged healing. Endodontic infection in DIO may exacerbate bone destruction in the long term *via chronically* elevating IL-1 signaling at a low level due to downregulation of *Il1rn*. However, the role of IL-1 signaling is diverse and complex. The impact of IL-1 signaling on both systemic and local conditions has not been fully understood. Thus, further studies are essential for the changes in IL-1 signaling associated with various systemic conditions, the underlying mechanisms, and infection-stimulated bone destruction.

## Figures and Tables

**TABLE 1:** The effect of deficiency/neutralization of each cytokine or receptor on periapical lesion.

	Cytokine/receptor	Effect on lesion size/abscess	IL-1 level in lesion	References
Neutralization	IL-1β^a^	Down		(95)
	IL-1α and IL-1β	Abscess formation	N.S.	(52)
Deficiency	IL-1R1	Up		(53, 54)
	TNF-α	N.S.		(65)
	IL-17A	Drastically down		(65)
	IL-17RA	Up	Up	(95)
	IFN-γ	N.S./up	N.S.	(65, 74, 75)
	IL-12	N.S.	N.S.	(74)
	IL-18	N.S.	N.S.	(74)
	IL-6	Up	Up	(69, 85)
	IL-4	N.S.	Down	(75, 91)
	IL-10	Drastically up	Drastically up	(75, 91)

a The effect of IL-1β neutralization was evaluated in IL-17RA^−/−^ model.Blank, not evaluated; N.S, no significant.

## Abbreviations

AP: apical periodontitis
DM: diabetes mellitus
DIO: diet-induced obesity
IFN-γ: gamma interferon
IL: interleukin
IL-1RA: interleukin-1 receptor antagonist
IL-1R1: interleukin-1 receptor type 1
IL-17RA: interleukin-17 receptor type A
MyD88: myeloid differentiation factor 88
NK cell: natural killer cell
OAF: osteoclast-activating factor
RANKL: receptor activator of NF-*κ*B ligand
T_reg_ cell: regulatory T cell
Th cell: thelper cell
TNF: tumor necrosis factor
